# O.3.3-8 Perspectives of neighborhood sport coaches towards using gamification to promote physical activity in the neighborhood

**DOI:** 10.1093/eurpub/ckad133.163

**Published:** 2023-09-11

**Authors:** Ayla Schwarz, Kirsten Verkooijen, Emely de Vet, Monique Simons

**Affiliations:** Wageningen University and Research, The Netherlands; ayla.schwarz@wur.nl; Wageningen University and Research, The Netherlands; ayla.schwarz@wur.nl; Wageningen University and Research, The Netherlands; ayla.schwarz@wur.nl; Wageningen University and Research, The Netherlands; ayla.schwarz@wur.nl

## Abstract

**Purpose:**

Gamification can be effective in promoting physical activity and can be applied in both physical and virtual environments. In the Netherlands, neighborhood sport coaches are intermediaries that play an important role in promoting physical activity in local communities. While gamification activities hold promise for promoting physical activity, gamification is only sporadically implemented by neighborhood sport coaches. This study aimed to assess the perceived barriers and facilitators among Dutch neighborhood sport coaches towards using gamification in their daily work.

**Methods:**

Twenty-two semi-structured interviews were conducted with neighborhood sport coaches in the Netherlands. The interviews were audiotaped, transcribed, and analyzed by means of thematic analysis using Atlas.ti 22 software. The analysis was informed by the COM-B model and the Theoretical Domains Framework.

**Results:**

Applying gamification to existing or new activities was highly valued by the neighborhood sport coaches. Themes that were identified related to support (i.e. in- and outside employer’s organization; established networks), skills (i.e. one’s level of technical or creative proficiency), knowledge (i.e. how to find gamified activities; proven effects), costs (i.e. high product costs; financial subsidies), time (i.e. time investment and prioritization), implementation (i.e. concerns about the digital divide; integration versus extension to current programs), and responsibility (i.e. within employer’s organization to promote gamification).

**Conclusions:**

This study outlines the need to strategically implement gamification in the work of neighborhood sport coaches by involving the employer’s organization or municipality. Establishing a gamification network among neighborhood sport coaches could facilitate the implementation and support neighborhood sport coaches to translate best practices to their local offer.

